# High electron mobility in strained GaAs nanowires

**DOI:** 10.1038/s41467-021-27006-z

**Published:** 2021-11-17

**Authors:** Leila Balaghi, Si Shan, Ivan Fotev, Finn Moebus, Rakesh Rana, Tommaso Venanzi, René Hübner, Thomas Mikolajick, Harald Schneider, Manfred Helm, Alexej Pashkin, Emmanouil Dimakis

**Affiliations:** 1 Institute of Ion Beam Physics and Materials Research, Helmholtz-Zentrum Dresden-Rossendorf, 01328 Dresden, Germany; 2 Center for Advancing Electronics Dresden (cfaed), Technische Universität Dresden, 01062 Dresden, Germany; 3 NaMLab gGmbH, Dresden, Germany

**Keywords:** Electronic devices, Nanowires, Nanowires

## Abstract

Transistor concepts based on semiconductor nanowires promise high performance, lower energy consumption and better integrability in various platforms in nanoscale dimensions. Concerning the intrinsic transport properties of electrons in nanowires, relatively high mobility values that approach those in bulk crystals have been obtained only in core/shell heterostructures, where electrons are spatially confined inside the core. Here, it is demonstrated that the strain in lattice-mismatched core/shell nanowires can affect the effective mass of electrons in a way that boosts their mobility to distinct levels. Specifically, electrons inside the hydrostatically tensile-strained gallium arsenide core of nanowires with a thick indium aluminium arsenide shell exhibit mobility values 30–50 % higher than in equivalent unstrained nanowires or bulk crystals, as measured at room temperature. With such an enhancement of electron mobility, strained gallium arsenide nanowires emerge as a unique means for the advancement of transistor technology.

## Abstract (web summary)

Semiconductor nanowires are promising candidates for the realization of novel transistor concepts. Here, the authors demonstrate that electron mobility in strained coaxial nanowire heterostructures can be higher than in the corresponding bulk crystals.

## Introduction

Many transistor concepts that aim at further miniaturisation, faster switching, lower power consumption or quantum transport applications rely on the nanowire or nanosheet geometry. Nanowires or nanosheets are inherently suitable for the realisation of gate-all-around field-effect transistors (FETs) that allow for the best possible electrostatic control of the channel potential. Various FET concepts like conventional inversion channel metal-oxide-semiconductor FETs^1,2^, junctionless FETs^3^, reconfigurable Schottky-barrier FETs^4^, band-to-band tunnel FETs (for steep current–gate-voltage subthreshold transfer characteristics below 60 mV/dec)^5^ or modulation-doped FETs^6^ can benefit from the gate-all-around architecture. Furthermore, very thin or core/shell heterostructured nanowires provide charge carrier confinement that leads to one-dimensional ballistic transport^7–10^, with the possibility to host multiple conduction channels within the same nanowire^11^. All the aforementioned devices also have the potential for monolithic integration in heterogeneous platforms like the mainstream Si CMOS^12^ or future platforms that may be based on emerging 2D materials^13^.

Owing to the high mobility of electrons in III–V semiconductors, this class of materials is the ideal candidate for high-speed low-power logic and radio-frequency applications. However, the mobility of charge carriers in nanowires made of III–V semiconductors is negatively affected by scattering at the interface of the nanowire to the surrounding isolation layers of the nanowires. Consequently, nanowires with smaller diameters, i.e. larger surface-to-volume ratios, exhibit lower mobility values^14,15^. To reduce interface scattering, the charge carriers are kept away from the nanowire surface by overgrowing the nanowires with a thick enough and usually lattice-matched shell of a semiconductor with a larger bandgap. With such an approach, electron mobility values of up to 3000 cm^2^ V^−1^ s^−1^ at room temperature for electron concentrations of 10^17^– 10^18^ cm^−3^ have been reported for the core of GaAs/Al_*x*_Ga_1-*x*_As core/shell nanowires, approaching values, which are typical for bulk GaAs^16^. Recently, we demonstrated that in GaAs/In_*x*_Al_1-*x*_As core/shell nanowires with large lattice mismatch between the core and the shell (the larger the In-content *x* in the shell, the larger the core/shell lattice mismatch), the few-μm-long core can be hydrostatically strained with remarkable effects on its electronic properties^17^. Specifically, hydrostatic tensile strain (expansion in all three dimensions) in the GaAs core of up to 7% was obtained, narrowing the bandgap of GaAs from its unstrained value of 1.42 eV at 300 K down to 0.87 eV (i.e. a reduction of 40%). Band structure calculations for hydrostatically tensile-strained GaAs predict that the effective mass of electrons in the Γ-valley of the conduction band should also undergo a significant reduction^17,18^. This implies higher electron mobility, which would be beneficial for high-speed low-power transistors. It should be noted that unlike biaxially strained thin films or small-sized asymmetrically shaped quantum dots, only nanowires offer the possibility for large hydrostatic strain in a transistor-relevant geometry. Improved mobility has been reported for holes in the core of [110]-oriented Ge/Si core/shell nanowires^19^, but that was attributed more to the high structural quality of the coherent heterostructure rather than the built-in strain (which was not hydrostatic in this case). A relative improvement of mobility due to strain has only been reported for electrons in the shell of Si_*x*_Ge_1-*x*_/Si core/shell nanowires^20^, but the actual mobility values were significantly lower than those in bulk Si.

Here, the transport properties of electrons inside the strained core of GaAs/In_0.37_Al_0.63_As core/shell nanowires are investigated to verify the aforementioned expectations and to assess the potential of this type of nanowires for transistor applications. First, the strain in the core and its effect on band structure are determined by a combination of spectroscopic methods and theoretical simulations. Then, the main study uses optical-pump THz-probe spectroscopy (OPTPS), which is a contactless method for probing the charge carrier transport and dynamics, circumventing challenges in the fabrication of electrical contacts on nanowires and the analysis of the corresponding electrical measurements^21^. Particular attention is paid to the role in OPTPS analysis of the spatial arrangement of the nanowires in the probed sample and the presence of an optional oxidation-protective In_*y*_Ga_1-*y*_As capping shell. The results of electron scattering rate and mobility are compared with those of unstrained GaAs/Al_0.35_Ga_0.65_As nanowires and bulk GaAs, revealing the beneficial effect of tensile strain. Electron mobility values higher than those in bulk crystals are demonstrated, raising the promises of mismatched core/shell nanowires for high-speed low-power transistor applications.

## Results

### Nanowire samples: general description and simulations

**Fig. 1:**
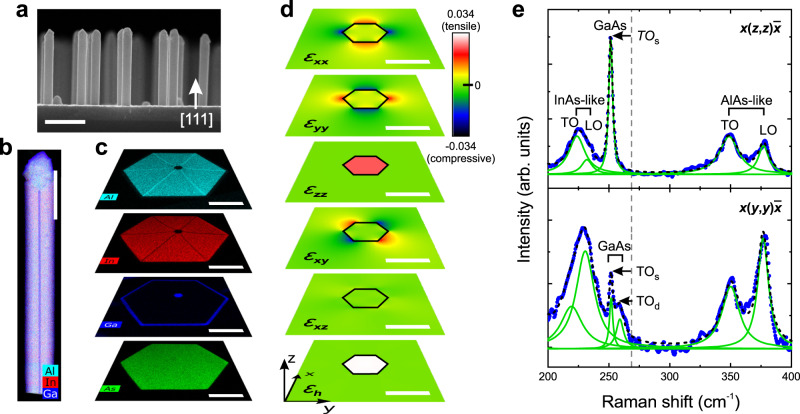
Morphology, chemical composition and strain of the investigated GaAs/In_0.37_Al_0.63_As core/shell (uncapped) and GaAs/In_0.43_Al_0.57_As/In_0.44_Ga_0.56_As core/shell/capping-shell (capped) nanowires. **a** Side-view scanning electron microscopy (SEM) image of as-grown capped nanowires on a Si(111) substrate. **b** Energy-dispersive X-ray spectroscopy (EDXS)-based compositional map from the side of one capped nanowire. The signal from Al, In and Ga is illustrated in cyan, red and blue, respectively. **c** Individual EDXS-based compositional maps for each constituent element perpendicular to the axis of a capped nanowire. Here, a TEM lamella from the middle of a nanowire was analysed. The signal from As is illustrated in green. **d** Simulated strain components close to the core of an uncapped nanowire using continuum elasticity theory. The core is outlined with a black line. **e** Raman scattering spectra at room temperature from a single uncapped nanowire in two different polarisation configurations: *x*(*z*,*z*)*x̅* at the top, and *x*(*y*,*y*)*x̅* at the bottom. The measured data were plotted with blue points and the fitted curves with lines: green for the individual peaks and dashed black for the cumulative curve. The scale bars correspond to 1 µm in (**a**), 400 nm in (**b**), 60 nm in (**c**) and 20 nm in (**d**).

This work was based on the study of two samples with free-standing GaAs/In_*x*_Al_1-*x*_As core/shell nanowires of zinc blende crystal structure, which were grown on Si(111) substrates by solid-source molecular beam epitaxy (MBE). The GaAs core was 2 µm long and 22 nm thick, whereas the In_*x*_Al_1-*x*_As shell was 80 nm thick. The main difference between the two samples is that for one of them, the In_*x*_Al_1-*x*_As shell was overgrown with a 5-nm-thick lattice-matched In_*y*_Ga_1-*y*_As capping shell (*y* ≈ *x* + 0.01) to protect the Al-containing shell from oxidation in air. The In-content *x* in the In_*x*_Al_1-*x*_As shell was in the range of 0.4 for both samples, i.e. 0.37 for the uncapped and 0.43 for the capped nanowires. More details about the growth and the structural properties of the nanowires can be found in ref. ^17^. A representative side-view scanning electron microscopy (SEM) image of the as-grown capped nanowires is shown in Fig. 1a. Chemical composition analysis was performed along and normal to the axis of single nanowires by energy-dispersive X-ray spectroscopy (EDXS) in a transmission electron microscope (TEM). Figure 1b, c shows representative EDXS-based element maps from capped nanowires. It can be seen that the core and the two shells are well defined and have uniform chemical compositions (except for the six In-poor corner lines along the $\left\langle 11\bar{2}\right\rangle$ crystallographic directions, which have been attributed to the combination of different growth rates, surface energies and possibly strain relaxation mechanisms on $\left\{1\bar{1}0\right\}$ and $\left\{11\bar{2}\right\}$ facets, as well as to the different diffusivities of In, Al and Ga adatoms^22^). The uncapped nanowires show the same morphological and compositional characteristics like the capped ones (see Supplementary Note 1). The measured core radius and In-content *x* in the shell are found to vary as little as ±2 nm (in agreement to previous SEM measurements on bare GaAs nanowires^23^) and ±0.02, respectively, within single nanowires or in different nanowires from the same sample (see Supplementary Note 1).

The strain distribution in the nanowires was simulated using three-dimensional finite-element continuum elasticity theory (nextnano^24^ software). Figure 1d shows selected results close to the core (at the middle of the nanowire length) for a nanowire with *x* = 0.37. The presence of a thin capping shell does not have any measurable influence on the strain. As it can be seen, both the axial (*ε*_zz_) and the radial (*ε*_xx_, *ε*_yy_) strain components inside the core are positive and quite uniform, with the axial one being the dominant one and practically equal to the lattice misfit (i.e. the relative difference between the lattice constants of the core and the shell). This means that the core is tensile-strained in all three dimensions and mostly along the nanowire axis. The large positive value of hydrostatic strain (*ε*_h_ = *ε*_zz_ + *ε*_xx_ + *ε*_yy_) expresses the significant volumetric increase of the crystal lattice. On the other hand, the strain components *ε*_xy_ and *ε*_xz_ (as well as *ε*_yz_, which is similar to *ε*_xz_) are equal to zero in the core. The values of all non-zero strain components at the centre of the core of the nanowires with *x* = 0.37, as well as for *x* = 0.43 (like the capped nanowires), are listed in Table 1. Concerning the strain in the shell, *ε*_zz_, *ε*_h_ and *ε*_xz_ are equal to zero. Only *ε*_xx_, *ε*_yy_ and *ε*_xy_ have complex non-zero patterns close to the interface with the core, but they all become equal to zero within ~10–20 nm away from it. This means that the shell can mainly be considered unstrained, which is understood as the result of its larger volume compared to that of the core^17^. The corresponding simulated distribution of stress in the core and the shell, together with simulations for the capped nanowires with *x* = 0.43, can be found in Supplementary Note 2.

**Table 1:** Simulated and measured strain components in the core of two types of GaAs/In_*x*_Al_1-*x*_As core/shell nanowires.

	Nanowire type	
	uncapped	capped
In-content *x*	0.37	0.43
Core/shell misfit	0.027	0.032
Simulated *ε*_zz_	0.025	0.029
Simulated *ε*_xx_, *ε*_yy_	0.004	0.005
Simulated *ε*_h_	0.033	0.038
Measured *ε*_zz_	0.027 (2)	0.031 (2)
Measured *ε*_xx_, *ε*_yy_	0.004 (1)	0.005 (1)
Measured *ε*_h_	0.035 (2)	0.041 (2)

The simulations employed continuum elasticity theory and the listed values correspond to the centre of the core. The measured values resulted from Eqs. 2 and 3. The x, y and z directions are defined in Fig. 1d. The positive values of all strain components indicate that the core is tensile-strained in all three dimensions. The small value of *ε*_xx_/*ε*_zz_ ratio indicates that the core is predominantly strained along the [111] nanowire axis. The In-content *x* (as measured by energy-dispersive x-ray spectroscopy) and the corresponding core/shell misfit are also listed.

Experimentally, the strain can be probed by the shift that it causes to the phonon lines of the core and the shell in micro-Raman scattering spectroscopy. Room temperature measurements were performed in the back-scattering configuration on single nanowires, which had been previously transferred on an Au-coated Si wafer, lying on their $\left\{1\bar{1}0\right\}$ side. Excitation (*λ* = 532 nm, beam spot size ≈1 μm) and measurements were performed normal to the Si wafer, thus normal to one of the $\left\{1\bar{1}0\right\}$ sidewalls of the nanowires (along x-axis), using a confocal microscope. The excitation light was linearly polarised with an orientation that could be arbitrarily rotated with respect to the nanowire axis (z-axis) in the (y, z) plane using a half-wavelength plate. For polarisation-resolved measurements, a rotatable analyser was placed in front of the detector. Figure 1e shows an example of Raman spectra from one uncapped nanowire in two polarisation configurations. The plot at the top shows measurement in *x*(*z*,*z*)*x̅* configuration, i.e. with excitation and measurement polarisation along the nanowire axis (z-axis). The peak at 252 cm^−1^ (249 cm^−1^ for the capped ones) corresponds to transverse optical (TO) phonons with atomic displacement along the nanowire axis (TO_s_) in the GaAs core. The downshift of the peak position with respect to ${\omega }_{{{{{{\rm{TO}}}}}}}$ = 268.6 cm^−1^ (indicated with a vertical dashed line) of bulk GaAs is attributed to tensile strain in the core. Transverse and longitudinal optical (LO) phonons from the shell are seen in the ranges of 220–230 cm^−1^ (InAs-like) and 350–375 cm^−1^ (AlAs-like). Their positions are close to those for bulk In_0.37_Al_0.63_As^25^, which is indicative of an almost strain-free shell. According to the selection rules for the specific polarisation configuration, only the TO phonons should be visible^26,27^. Nevertheless, the presence of LO modes can be attributed to possible geometric deviations (e.g. not completely planar nanowires), the finite detection angle, as well as the possible roughness of the shell surface. The Raman spectrum is similar in *x*(*y*,*y*)*x̅* configuration (bottom plot in Fig. 1e), i.e. with excitation and measurement polarisation orthogonal to the nanowire axis (along the y-axis). Besides TO_s_, though, GaAs TO phonons with atomic displacement along the y-axis (TO_d_) are visible at 259 cm^−1^. TO_s_ and TO_d_ are degenerate in bulk GaAs, whereas their splitting in the nanowire core stems from the strain anisotropy (shear strain)^26,28,29^.

In a previous study on the same type of nanowires, we considered only the role of volumetric strain in phonon shifts, neglecting the contribution of shear strain. Nevertheless, the deduced strain values from Raman scattering measurements could sufficiently explain the observed changes in the bandgap of the GaAs core in good agreement with theory^17^. Here, both volumetric and shear contributions are taken into account for a more precise analysis. Assuming uniform strain around the [111] axis, the strain tensor in Cartesian coordinates gets the form^30^

1 $${{{{{\boldsymbol{\varepsilon }}}}}}=\frac{1}{3}\left(\begin{array}{ccc}2{\varepsilon }_{{{{{{\rm{xx}}}}}}}+{\varepsilon }_{{{{{{\rm{zz}}}}}}} & {\varepsilon }_{{{{{{\rm{zz}}}}}}}-{\varepsilon }_{{{{{{\rm{xx}}}}}}} & {\varepsilon }_{{{{{{\rm{zz}}}}}}}-{\varepsilon }_{{{{{{\rm{xx}}}}}}}\\ {\varepsilon }_{{{{{{\rm{zz}}}}}}}-{\varepsilon }_{{{{{{\rm{xx}}}}}}} & 2{\varepsilon }_{{{{{{\rm{xx}}}}}}}+{\varepsilon }_{{{{{{\rm{zz}}}}}}} & {\varepsilon }_{{{{{{\rm{zz}}}}}}}-{\varepsilon }_{{{{{{\rm{xx}}}}}}}\\ {\varepsilon }_{{{{{{\rm{zz}}}}}}}-{\varepsilon }_{{{{{{\rm{xx}}}}}}} & {\varepsilon }_{{{{{{\rm{zz}}}}}}}-{\varepsilon }_{{{{{{\rm{xx}}}}}}} & 2{\varepsilon }_{{{{{{\rm{xx}}}}}}}+{\varepsilon }_{{{{{{\rm{zz}}}}}}}\end{array}\right)$$

where the diagonal (off-diagonal) elements represent the hydrostatic (shear) components. The frequencies of TO_s_ (${\omega }_{{{{{{{\rm{TO}}}}}}}_{s}}$) and TO_d_ (${\omega }_{{{{{{{\rm{TO}}}}}}}_{{{{{{\rm{d}}}}}}}}$) in the presence of strain can be found by solving the corresponding secular equation as described in ref. ^31^. In this way, one gets^30,31^

2 $${{\omega }_{{{{{{{\rm{TO}}}}}}}_{{{{{{\rm{s}}}}}}}}}^{2}={{\omega }_{{{{{{\rm{TO}}}}}}}}^{2}\left[1-2{\gamma }_{{{{{{\rm{T}}}}}}}\left(2{\varepsilon }_{{{{{{\rm{xx}}}}}}}+{\varepsilon }_{{{{{{\rm{zz}}}}}}}\right)+\frac{4}{3}{\widetilde{K}}_{44}^{{{{{{\rm{T}}}}}}}\left({\varepsilon }_{{{{{{\rm{zz}}}}}}}-{\varepsilon }_{{{{{{\rm{xx}}}}}}}\right)\right],$$

3 $${{\omega }_{{{{{{{\rm{TO}}}}}}}_{{{{{{\rm{d}}}}}}}}}^{2}={{\omega }_{{{{{{\rm{TO}}}}}}}}^{2}\left[1-2{\gamma }_{{{{{{\rm{T}}}}}}}\left(2{\varepsilon }_{{{{{{\rm{xx}}}}}}}+{\varepsilon }_{{{{{{\rm{zz}}}}}}}\right)-\frac{2}{3}{\widetilde{K}}_{44}^{{{{{{\rm{T}}}}}}}\left({\varepsilon }_{{{{{{\rm{zz}}}}}}}-{\varepsilon }_{{{{{{\rm{xx}}}}}}}\right)\right],$$

where ${\omega }_{{{{{{\rm{TO}}}}}}}$ = 268.6 cm^−1^ is the TO phonon frequency for strain-free GaAs, ${\gamma }_{{{{{{\rm{T}}}}}}}$ = 1.35 is the hydrostatic deformation potential (mode Grüneisen parameter), and ${\widetilde{K}}_{44}^{{{{{{\rm{T}}}}}}}$ = −0.88 is the shear deformation potential for GaAs TO phonons^29^. The second (third) term inside the square bracket represents the contribution of hydrostatic (shear) strain to the total phonon shift. One can see that the hydrostatic strain causes the same shift for both ${\omega }_{{{{{{{\rm{TO}}}}}}}_{{{{{{\rm{s}}}}}}}}$ and ${\omega }_{{{{{{{\rm{TO}}}}}}}_{{{{{{\rm{d}}}}}}}}$, whereas the shear strain is responsible for the splitting between the two frequencies. From the measured values of ${\omega }_{{{{{{{\rm{TO}}}}}}}_{{{{{{\rm{s}}}}}}}}$ = 251(1) cm^−1^ and ${\omega }_{{{{{{{\rm{TO}}}}}}}_{{{{{{\rm{d}}}}}}}}$ = 258(1) cm^−1^ on five uncapped nanowires, the strain components in the GaAs core were calculated from Eqs. 2 and 3 to be *ε*_zz_ = 0.030(6), *ε*_xx_ = 0.002(2) and *ε*_h_ = 0.034(8). Despite of a slight overestimation (underestimation) of *ε*_zz_ (*ε*_xx_), the values are in reasonable agreement with the results from elasticity theory in Table 1. Because of the low intensity of the TO_d_ peak, though, it was difficult to repeat the measurement of ${\omega }_{{{{{{{\rm{TO}}}}}}}_{{{{{{\rm{d}}}}}}}}$ on many nanowires. For this reason, the statistical sampling relied exclusively on measurements of ${\omega }_{{{{{{{\rm{TO}}}}}}}_{s}}$ and the use of Eq. 2, knowing from elasticity theory that *ε*_xx_ ≈ 0.15 *ε*_zz_. The results for both uncapped and capped nanowires are listed in Table 1. It is worthwhile to mention that owing to the three-dimensional stress in the core, the ratio of - *ε*_xx_/*ε*_zz_ is negative, in contrast to the positive Poisson’s ratio of 0.16 for uniaxially stressed GaAs nanowires^26^.

**Fig. 2:**
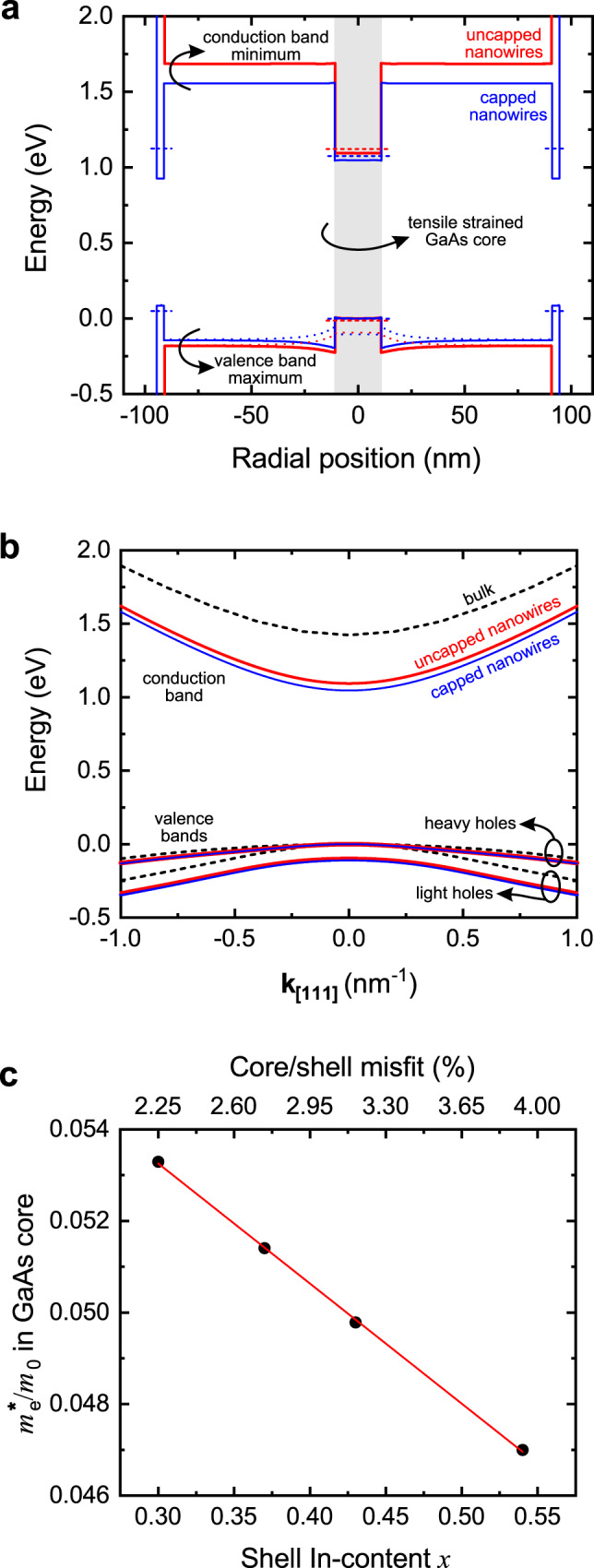
Simulated electronic structure of the investigated GaAs/In_0.37_Al_0.63_As core/shell (uncapped) and GaAs/In_0.43_Al_0.57_As/In_0.44_Ga_0.56_As core/shell/capping-shell (capped) nanowires at 300 K using 8-band **k**·**p** theory. **a** Band-edge profile at the Γ-point of the Brillouin zone, along the $\left[1\bar{1}0\right]$ crystallographic direction (from the middle of one side-facet, through the centre of the core at zero position, to the middle of the opposite side-facet). The valence bands for heavy and light holes are shown with continuous and dotted lines, respectively. The lowest confined energy levels in the core and the capping shell are indicated with horizontal dashed lines. **b** Γ-valley energy dispersion in the GaAs core, simulated along the [111] crystallographic orientation. One conduction and two valence bands (heavy and light holes) are shown. Results for unstrained bulk are also shown (black dashed lines) for comparison. The valence band maximum at **k**_**[111]**_ = 0 has been set to zero energy. **c** Simulated dependence of the electron effective mass in the GaAs core on the In-content *x* of the In_*x*_Al_1-*x*_As shell (bottom x-axis) and the corresponding core/shell misfit (top x-axis). The data points are calculated values, which have been linearly fitted.

It is well known that the hydrostatic strain in GaAs changes the bandgap, whereas the shear strain separates the valence bands of heavy and light holes at the Γ-point of the Brillouin zone, lifting the degeneracy that characterises the unstrained GaAs^17,18,26,32^. Here, the optical bandgap of the tensile-strained core for both uncapped and capped nanowires was measured by photoluminescence (PL) spectroscopy to be in the range of 1.1 eV at room temperature (to be discussed in more detail in the following), i.e. ~0.3 eV lower than the expected value for unstrained GaAs (1.453 eV) assuming the same radial confinement. These values are in good agreement with our 8-band **k**·**p** calculations (using nextnano) shown in Fig. 2a, where the simulated band-edge profiles at the Γ-point of the Brillouin zone across the core/shell heterostructure at 300 K for both samples are plotted. The core of the uncapped (capped) nanowires has an optical bandgap of 1.136 eV (1.071 eV) and is surrounded by the shell of 1.862 (1.695) eV. The capping shell has been considered to have an effective thickness of 3 nm after oxidation of the outer 2 nm, which results in an optical bandgap of 1.075 eV. The calculations were performed along the $[1\bar{1}0]$ crystallographic direction, i.e. normal to the nanowire sidewalls and through the core centre (zero position).

Besides bandgap narrowing, a reduction of the electron effective mass in the tensile-strained core is also expected^18^. Our calculations shown in Fig. 2b (energy dispersion around the Γ-point) predict a reduction of the effective mass of electrons from 0.067 *m*_0_ for unstrained GaAs to 0.0514 *m*_0_ (0.0498 *m*_0_) for the strained GaAs core in the uncapped (capped) nanowires. The general dependence of the electron effective mass in the GaAs core on the In-content of the In_*x*_Al_1-*x*_As shell and the corresponding core/shell misfit is plotted in Fig. 2c. In the following, we will show that electrons in the strained core exhibit high mobility values, well above the previously reported ones for unstrained GaAs nanowires, as well as for bulk GaAs.

### Electron transport properties

**Fig. 3:**
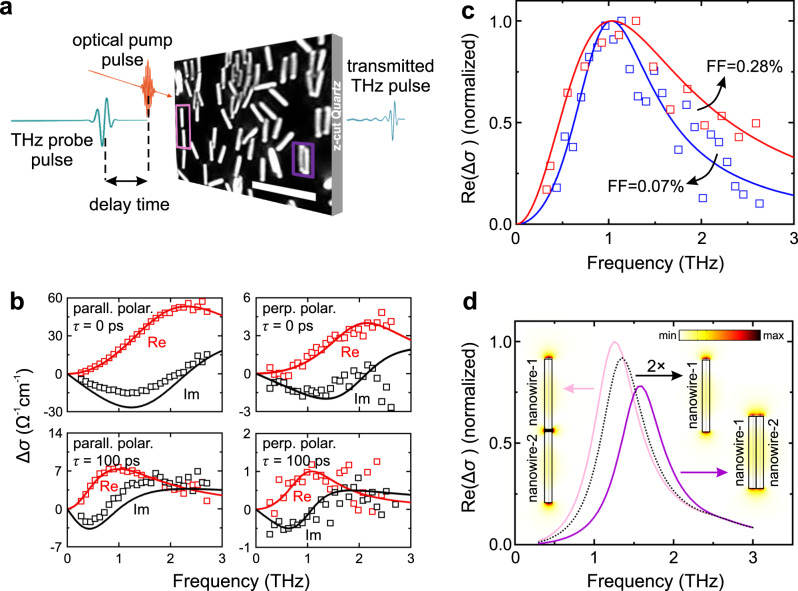
Basic description and characteristics of optical-pump THz-probe spectroscopy measurements on core/shell nanowires. **a** Schematic description of the basic principle of the method. All measurements were performed at room temperature. The optical microscopy image shows transferred nanowires on z-cut quartz and was acquired with 100× magnification in dark-field mode. Two examples of nanowires in close proximity are indicated with pink (nanowires in a row) and purple (nanowires side-by-side) frames. The scale bar corresponds to 5 μm. **b** Photoconductivity spectra from capped GaAs/In_0.49_Al_0.51_As nanowires at selected pump-probe delays (0 and 100 ps) and different polarisations of the THz-probe pulse (parallel and perpendicular to the nanowires). The real part is shown in red symbols and the imaginary part in black symbols. The lines are fits to the data using the localised surface plasmon (LSP) model described in Eqs. 4 and 5. **c** Real part of the photoconductivity spectra (*τ* = 70 ps) from two areas with the different number density of capped GaAs/In_0.43_Al_0.57_As nanowires on the same sample. The corresponding filling factor (FF) was 0.28% (red symbols) and 0.07% (blue symbols). The lines are LSP model fits. **d** Calculated (by COMSOL) real part of the photoconductivity spectra of two distant (black curve) or two grouped photo-doped GaAs/In_0.44_Al_0.56_As nanowires in two different configurations (pink and purple curves), as shown in the insets. The spectra are normalised to the highest value. The two configurations of grouped nanowires resemble those of the framed nanowires in (**a**). The simulated component of the THz-probe electric field (modulus) along the nanowire axis is also shown in the insets as a colour plot. The colour scale ranges from 0 to 10 V m^−1^ (the probe field was set to be 1 V m^−1^ far away from the nanowires).

The transport properties of electrons in the core/shell nanowires were probed at room temperature by OPTPS. The particular method has been established in recent years as a reliable way to measure electron transport and dynamics in nanowires^21,33–35^. The basic principle is schematically described in Fig. 3a. Nanowires were transferred from their Si substrate onto z-cut quartz, which is transparent for the THz radiation in the range of 0–3.8 THz^14^. The transfer method (rubbing of quartz against the as-grown sample) aimed at obtaining lying nanowires on quartz, with their axes oriented along the same direction. As it is shown in the dark-field optical microscopy image in Fig. 3a, this was roughly the case for the majority of the nanowires, but not for all of them (see Supplementary Note 3). Nevertheless, we will be referring to the average orientation of the majority of the nanowires in the following for the description of our measurements and analyses. An optical-pump pulse with an average photon energy of 1.55 eV, pump fluence of 70 μJ cm^−2^, pulse duration of 60 fs and polarisation parallel to the nanowire axes generates electrons and holes only inside the GaAs core and the In_0.44_Ga_0.56_As capping shell (multi-photon generation of charge carriers in the In_*x*_Al_1-*x*_As shell for *x* = 0.37 or 0.43 is negligible). After a delay time *τ*, THz pulses in the frequency range of *ω*/2π = 0.2–2.7 THz, with polarisation that can be set either parallel or perpendicular to the nanowire axes, are transmitted through the sample, driving plasmon oscillations of the photo-generated carriers. Time-domain THz spectroscopy allows for measuring the complex conductivity spectrum Δ*σ*(*ω*,*τ*), which carries information about the dynamics of the average density *n*(*τ*) and the average mobility *μ*_e_(*τ*) of the photo-generated electrons in the probed ensemble of nanowires (the contribution from the much heavier holes is neglected). Comparative measurements on uncapped and capped nanowires allowed us to distinguish between the contributions from electrons in the core and the capping shell. More details about the OPTPS setup are given in Methods.

Figure 3b shows the complex photoconductivity spectra Δ*σ*(*ω*) for two selected time delays (0 and 100 ps) and for two polarisation directions of the THz pulse (parallel and perpendicular to the nanowire axes). The characteristic peak in the real part of Δ*σ*(*ω*) is the signature of localised surface plasmons (LSP) in the nanowires. For both delay times, the conductivity signal for the parallel THz polarisation is almost one order of magnitude higher than that for the perpendicular one. This result manifests that only the lowest-order longitudinal LSP mode along the [111] nanowire axis is excited by the THz probe, in agreement with the previous reports^14,33,35,36^. This is true independent of the presence of a capping shell or the actual value of *x* (here, an auxiliary sample with capped nanowires with *x* = 0.49 was measured). The sensitivity only to the longitudinal LSP mode means that the nanowires that are not exactly parallel to the THz polarisation will just contribute less to the conductivity signal^35^.

The conductivity spectra Δ*σ*(*ω*) at a given *τ* can be fitted with an LSP model (the contribution of unintentional dopants is neglected) using a Lorentzian function of the form^33^

4 $$\Delta \sigma \left(\omega \right)=\frac{{in}{e}^{2}\omega }{{m}_{{{{{{\rm{e}}}}}}}^{\ast }\left({\omega }^{2}-{\omega }_{0}^{2}+i\omega \gamma \right)},$$

where the electron density $n$ is equal to

5 $$n=\frac{{m}_{{{{{{\rm{e}}}}}}}^{\ast }{\varepsilon }_{{{{{{\rm{r}}}}}}}{\varepsilon }_{0}}{g{e}^{2}}{\omega }_{0}^{2}.$$

In these equations, *ω*_0_ is the surface plasmon resonance frequency (i.e. the peak position of Re Δ*σ*(*ω*)), *γ* is the momentum scattering rate of electrons (which is associated with the peak width of Re Δ*σ*(*ω*)), ${m}_{{{{{{\rm{e}}}}}}}^{\ast }$ is the electron effective mass, *e* is the electronic charge, g is the geometrical factor and *ε*_r_ = 14.16 and *ε*_0_ is the electric permittivity of the strained nanowire core and the free space in the THz range, respectively^37–39^. The fitted conductivity spectra are shown with continuous curves in Fig. 3b, where *ω*_0_ and *γ* are the only fitting parameters. Finally, the electron mobility *μ*_e_ is calculated from ref. ^33^

6 $${\mu }_{{{{{{\rm{e}}}}}}}=\frac{e}{{m}_{{{{{{\rm{e}}}}}}}^{\ast }\gamma }.$$

The model assumes that the peak width of Re Δ*σ*(*ω*) is dictated only by the value of *γ*. In practice though, we have observed that the peak can be additionally broadened when ensembles of nanowires with a higher number density are measured (for a higher signal-to-noise ratio), where inevitably some of the nanowires are in close proximity or even in contact with others. An example is shown in Fig. 3c (capped nanowires with *x* = 0.43), where measurements performed on two locations with different number density of nanowires on the same sample are compared (for the same carrier concentration; *τ* = 70 ps). The peak width of Re Δ*σ*(*ω*) is 70% larger for measurements on the dense area (filling factor FF = 0.28%, 1.84 THz) compared to those on the sparse one (FF = 0.07%, 1.08 THz). Consequently, the deduced mobility from Eq. 6 appears to be 41% lower in the dense area. This means that the extracted electron mobility from OPTPS can generally be regarded as a lower limit for the real value of an isolated nanowire. The accuracy of the method improves for samples with lower number densities of nanowires, where the probability to have nanowires in close proximity or in contact with each other is lower.

If two or more nanowires are very close or even touch each other, they form different geometries that generally have different plasmon resonances. To analyse the effect of the nanowire proximity on the conductivity spectra, we numerically modelled the plasmonic response of two photo-doped GaAs/In_0.44_Al_0.56_As NWs in an external electric field (which plays the role of a THz-probe) using COMSOL. The two nanowires were positioned in three simple configurations (shown as insets in Fig. 3d): in a row along their axes, side-by-side, or in large distance (isolated) from each other. As shown in Fig. 3d, if the two nanowires are positioned in a row, the simulated peak position is shifted to lower frequencies (pink curve) compared to the isolated nanowires (black dotted curve). In contrast, if the nanowires are positioned side-by-side, the peak position is shifted to higher frequencies (purple curve). In Fig. 3a, examples of grouped nanowires that are similar to the simulated configurations are framed with the corresponding colours. Considering that OPTPS measurements are performed on large ensembles of randomly positioned nanowires, where isolated and grouped nanowires coexist, the Re Δ*σ*(*ω*) spectrum is expected to be inhomogeneously broadened owing to the superposition of peaks from both isolated and grouped nanowires. The arrangement and number density of nanowires cannot be measured at the exact location of OPTPS measurements. However, the number density can be estimated from the Δ*σ*(*ω*) intensity and the fitted FF or by using optical microscopy on the same area of the sample after the OPTPS measurements. Although the number density of nanowires alone does not strictly reflect the level of nanowire grouping, a larger inhomogeneous broadening can generally be expected for denser ensembles, in agreement with the results in Fig. 3c. Furthermore, the inhomogeneous broadening in Fig. 3c is more pronounced on the high-frequency wing of the LSP peak, suggesting a stronger contribution from nanowires that are grouped side by side.

**Fig. 4:**
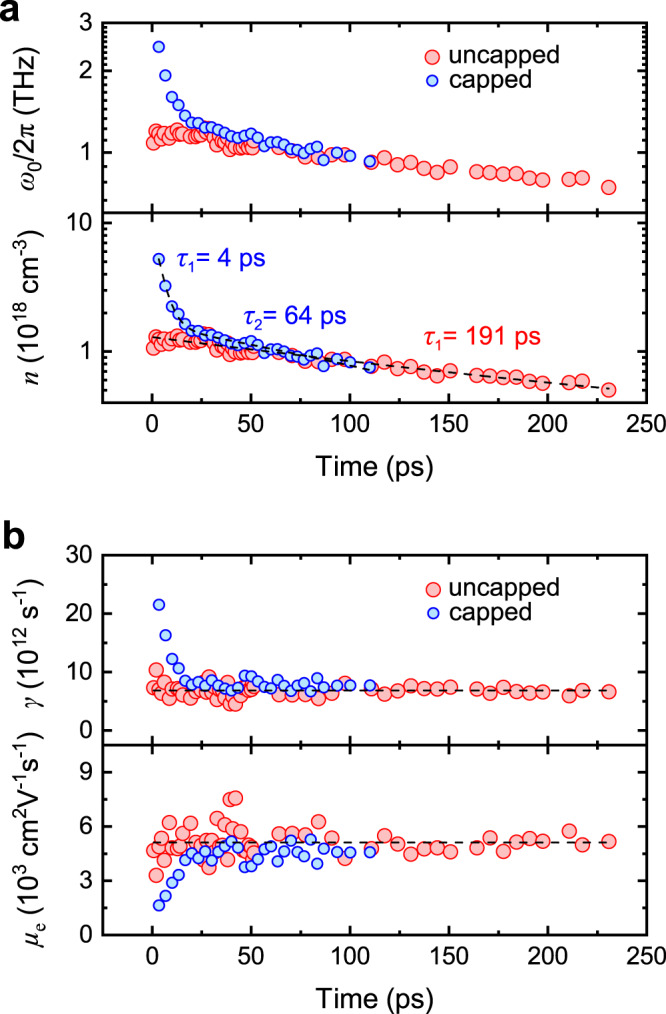
Analysis of electron transport dynamics inside GaAs/In_0.37_Al_0.63_As (uncapped) and GaAs/In_0.43_Al_0.57_As/In_0.44_Ga_0.56_As (capped) nanowires based on optical-pump THz-probe spectroscopy measurements at room temperature. **a** Temporal evolution of plasmon frequency *ω*_0_ (top) and the associated electron density *n* (bottom). The dashed lines are mono-exponential and bi-exponential fits for the uncapped and capped nanowires, respectively. **b** Temporal evolution of momentum scattering rate *γ* (top) and the associated mobility *μ*_e_ (bottom) of electrons. The dashed lines are linear fits for the uncapped nanowires.

The analysis of the conductivity spectra was repeated for different delay times and the results are shown in Fig. 4 for uncapped (red data points) and capped (blue data points) core/shell nanowires. The measurements were performed on relatively sparse ensembles of nanowires to minimise the inhomogeneous plasmon broadening effect. Unavoidably, the resulting signal-to-noise ratio was quite low even for the relatively high pump fluence of 70 µJ cm^−2^ that was used. Thus, a reliable analysis was possible only within the first few hundreds of ps of delay time. Figure 4a shows the monotonic decrease of *ω*_0_ and the corresponding *n* as a function of time, which is the result of the recombination of photo-generated electrons in the core of the uncapped nanowires or in both the core and the capping shell of the capped nanowires. An apparent difference between the two types of nanowires is the higher $n$ at zero delay time and its faster decay within the first 40 ps in capped nanowires. This is attributed to the photo-generation of electrons in the capping shell and, subsequently, their fast recombination at the free-surface states, similar to reports for GaAs/Al_0.4_Ga_0.6_As/GaAs core/shell/capping-shell nanowires^16^. After the first few tens of ps, the decay rate of $n$ in capped nanowires becomes slower, i.e. more representative of electrons in the core.

**Fig. 5:**
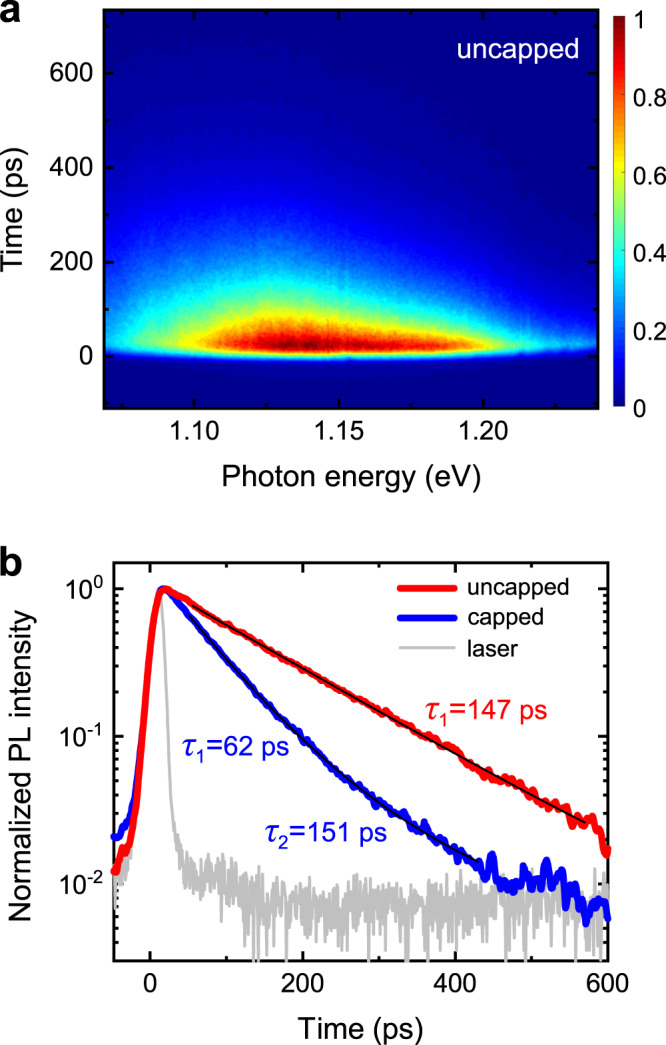
Study of charge carrier dynamics in the core of GaAs/In_0.37_Al_0.63_As (uncapped) and GaAs/In_0.43_Al_0.57_As/In_0.44_Ga_0.56_As (capped) core/shell nanowires at room temperature. **a** Time-resolved photoluminescence spectroscopy scan from uncapped nanowires. **b** Integrated photoluminescence intensity (normalised) as a function of time for uncapped (red curve) and capped (blue curve) nanowires. The data have been fitted with mono-exponential (uncapped nanowires) and bi-exponential (capped nanowires) decay functions (black curves), and the resulting lifetimes are indicated.

The experimental data for the uncapped nanowires are best fitted with a mono-exponential decay function, considering only monomolecular recombination, i.e. ${dn}/{dt}\propto n$. The electron lifetime is deduced to be 191 ps. In any case, bimolecular and Auger recombination in GaAs are negligible in the range of *n* = 10^17^–10^18^ cm^−3^ ^40^. Similar carrier dynamics were observed in time-resolved PL measurements. Figure 5a shows the measured spectra at room temperature with excitation energy of 1.55 eV. As shown in Fig. 5b (red curve), the integrated PL signal is also fitted as a mono-exponential decay with a lifetime of 147 ps.

According to previous reports^41,42^, electron dynamics in such thin nanowires is governed by recombination at the core/shell interface. The corresponding interface recombination velocity *S* for electrons in the core can be deduced from^43^

7 $$\frac{1}{{\tau }_{{{{{{\rm{core}}}}}}}}=\frac{1}{{\tau }_{{{{{{\rm{volume}}}}}}}}+\frac{2S}{R},$$

where *R* is the core radius, *τ*_core_ is the measured lifetime (191 ps by OPTPS or 147 ps by PL), and *τ*_volume_ is the time constant for recombination in the core excluding the effect of the core/shell interface. Using *τ*_volume_ = 1.6 – 2.5 ns according to reports on passivated unstrained GaAs nanowires^16,41^, *S* is estimated to be in the range of 2.8 – 4.0 × 10^3^ cm s^−1^. The fact that similar values have been reported for lattice-matched core/shell nanowires with GaAs core^41,44^ points to the high quality of the GaAs/In_0.37_Al_0.63_As interface despite of the large lattice mismatch. It should not be excluded though that the low value of *S* may also be a result of the saturation of interface traps under the particular pump fluence used here^42^.

The dynamics of electrons in the capped nanowires is more complex. OPTPS in Fig. 4a (blue points) combined with time-resolved PL in Fig. 5b (blue curve; see the full scan in Supplementary Note 4) reveal the existence of three distinct lifetimes of 4, 62–64 and 151 ps. It should be mentioned that the longest lifetime of 151 ps is not seen in OPTPS because of the restricted time range of 0–100 ps (the signal-to-noise ratio becomes too low after that because of the low density of the nanowire ensemble), whereas the shortest lifetime of 4 ps is not resolved in PL owing to the limited temporal resolution of the experimental setup. Concerning their origin, the lifetime of 151 ps is similar to that in uncapped nanowires and, thus, it is attributed to electrons in the strained GaAs core. On the other hand, the shorter lifetimes of 4 and 62–64 ps, which were not observed in uncapped nanowires, probably correspond to the capture of electrons in the In_0.44_Ga_0.56_As capping shell via saturable surface traps and non-saturable recombination channels, respectively^14,33^.

Figure 4b shows the dynamics of the scattering rate of electrons *γ*, as extracted from OPTPS measurements at different delay times, and the corresponding mobility *μ*_*e*_ from Eq. 6, where the previously calculated value of ${m}_{{{{{{\rm{e}}}}}}}^{\ast }$ (0.0514 *m*_0_ for uncapped and 0.0498 *m*_0_ for capped nanowires) was used for the strained core. As it is observed, electrons in the core of the uncapped nanowires exhibit quite stable values in the range of *γ* = 6.8 × 10^12^ s^−1^ and *μ*_e_ = 5100 cm^2^ V^−1^ s^−1^. On the other hand, a temporal variation is observed for the capped nanowires as a result of the combined contribution of electrons in the strained core and the capping shell. Owing to the strong electron scattering at the free surface of the capping shell, higher *γ* and, consequently, lower *μ*_e_ values are measured just after the pump pulse, i.e. for *τ* ≈ 0 (electrons with the same value of ${m}_{{{{{{\rm{e}}}}}}}^{\ast }$ in both the strained GaAs core and the unstrained In_0.44_Ga_0.56_As shell^45^ have been assumed). Within the first 40 ps, though, the population of electrons in the capping shell decreases fast (recall the short lifetimes) and the contribution of electrons in the core dominates, exhibiting *γ* and *μ*_e_ values similar to those in uncapped nanowires.

**Fig. 6:**
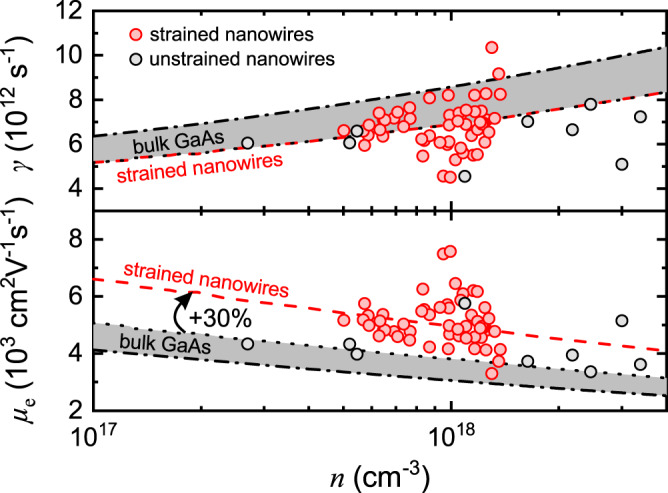
Momentum scattering rate *γ* (top) and the associated mobility *μ*_e_ (bottom) of electrons as a function of electron density *n* inside the strained core of uncapped GaAs/In_0.37_Al_0.63_As core/shell nanowires (red symbols) and the unstrained core of GaAs/Al_0.35_Ga_0.65_As/GaAs core/shell/capping-shell nanowires (grey symbols). All data originate from optical-pump THz-probe spectroscopy (OPTPS) measurements at room temperature. For comparison, OPTPS data (fitted by the Caughey-Thomas relation) for undoped bulk GaAs from refs. ^46^ (black dotted curve) and ^48^ (black dash-dotted curve) are also shown. The grey-shaded area covers all values between the two reference curves. The red dashed curves describe the results for strained nanowires.

Combining the data in Fig. 4a, b, the dependence of *γ* and the corresponding *μ*_e_ on *n* is deduced, as shown in Fig. 6 (red symbols). Here, only the results for uncapped nanowires, thus for electrons in the core, are shown. For comparison, OPTPS measurements were also performed on GaAs/Al_0.35_Ga_0.65_As/GaAs core/shell/capping-shell nanowires of similar dimensions (22 nm thick core, 80 nm thick shell, 5 nm thick capping shell). In this case, the core is unstrained and the results for the electrons therein are also shown in Fig. 6 (grey symbols). Furthermore, OPTPS data for bulk GaAs from the literature^46–48^ are also plotted (black dotted and dash-dotted lines; the data were either directly copied or calculated using Eq. 6). The shadowed area in the plot covers all values between those in ref. ^46^ (black dotted line) and ref. ^48^ (black dash-dotted line).

Concerning *γ* (top plot in Fig. 6), both strained and unstrained nanowires exhibit average values close to the lowest reported ones for bulk GaAs. This means that neither the decrease of electron effective mass in strained nanowires nor the thin diameter (large surface-to-volume ratio) of the core affect significantly, if at all, the total scattering rate of electrons. Let us now discuss what this means for the individual scattering mechanisms that are relevant here. Typically, the scattering of electrons by LO phonons (Fröhlich interaction) is the dominant scattering mechanism in undoped bulk GaAs at room temperature. Scattering rates of ~3 × 10^12^ s^−1^, which correspond to mobility values of ~8700 cm^2^ V^−1^ s^−1^, have been reported^49^. In OPTPS measurements, though, also the scattering of electrons by holes becomes important because equal densities of electrons and holes are photo-generated. This explains the relatively higher (lower) values of scattering rate (mobility), as well as their increase (decrease) with *n*, shown in Fig. 6 for bulk GaAs^16,47,48^. Furthermore, the scattering of electrons at the core/shell interface (by crystal defects, interface roughness, and other imperfections) may also come into play for nanowires with a thin core. In the end, the total scattering rate *γ* can be approximated by Matthiessen’s rule as^50^

8 $$\gamma ={\gamma }_{{{{{{\rm{p}}}}}}}+{\gamma }_{{{{{{\rm{h}}}}}}}+{\gamma }_{{{{{{\rm{i}}}}}}},$$

where *γ*_p_, *γ*_h_ and *γ*_i_ are the individual scattering rates of electrons by bulk LO phonons, photo-generated holes and the core/shell interface, respectively. Using the data for bulk GaAs (*γ* = 7–8 ×10^12^ s^−1^ and *γ*_p_ ≈ 3 × 10^12^ s^−1^)^46,48,49^ in Eq. 8, we obtain *γ*_h_ = 4–5 ×10^12^ s^−1^, i.e. comparable scattering rates by holes and LO phonons. Assuming that the same *γ*_p_ and *γ*_h_ values also exist in unstrained nanowires (confined or interface phonons are neglected for the particular core radius^51,52^), the similar total scattering rates found for unstrained nanowires and bulk GaAs suggest that the interface scattering is negligible: *γ*_i_ « *γ*_p_ + *γ*_h_. This result is reasonable for a longitudinal LSP mode because the transport of electrons is parallel to the core/shell interface. In tensile-strained nanowires, *γ*_p_ and *γ*_h_ should be affected as follows: the decrease of ${m}_{{{{{{\rm{e}}}}}}}^{\ast }$ from 0.067 to 0.0514 should result in a 12% decrease of *γ*_p_ compared to unstrained nanowires and bulk GaAs because *γ*_p_ is proportional to ${{m}_{{{{{{\rm{e}}}}}}}^{\ast }}^{1/2}$^49,53,54^. On the other hand, *γ*_h_ is expected to increase by a similar percentage because it is inversely proportional to the reduced mass of electrons and holes: ${{\gamma }_{{{{{{\rm{h}}}}}}}\propto \left({{m}_{{{{{{\rm{e}}}}}}}^{\ast }}^{-1}+{{m}_{{{{{{\rm{h}}}}}}}^{\ast }}^{-1}\right)}^{1/2}$ (as described by the equation for scattering by ionised impurities^47,54–56^). A further increase of *γ*_h_ could result from the higher concentration of heavy holes compared to light holes in strained nanowires because of the valence band splitting. After all, the effect of strain on the total scattering rate $\gamma$ must be limited because of the counterbalancing of the decreased *γ*_p_ by the increased *γ*_h_. Indeed, although the scattering of OPTPS data in Fig. 6 obscures any small changes in *γ*, no obvious dependence on strain can be resolved.

The corresponding mobility values *μ*_e_ are shown in the bottom plot of Fig. 6. The values for unstrained GaAs nanowires are the highest reported so far, which we consider a consequence of both the high structural quality of the nanowires and the minimisation of the inhomogeneous plasmon broadening in sparse nanowire ensembles, and agree well with the highest reported ones for bulk GaAs^46^. Most important though, the strained nanowires exhibit even higher values by a factor of 1.3 (this is equal to the effective mass ratio 0.067/0.0514). This is because they have similar *γ* but lower ${m}_{{{{{{\rm{e}}}}}}}^{\ast }$ compared to unstrained nanowires and bulk GaAs. It is remarkable that high average values in the range of 5500–4800 cm^2^ V^−1^ s^−1^ for electron densities of 5–14 × 10^17^ cm^−3^ are obtained for a core diameter as small as 22 nm, i.e. approximately only twice the exciton Bohr radius in GaAs. This implies a high structural quality of the core/shell interface and its minor influence on the electron transport along the nanowire axis. Furthermore, the electron mobility without the scattering of electrons by holes, which is an inherent effect of the photoexcitation in OPTPS, can be estimated from Eq. 6 after setting *γ* = (0.0514/0.067)^1/2^ ∙ *γ*_p_ (where *γ*_p_ ≈ 3 ×10^12^ s^−1^ is the value for bulk GaAs^49^). This results in *μ*_e_ in the range of 13,000 cm^2^/Vs, i.e. an increase of 50% with respect to bulk GaAs.

## Discussion

Our results demonstrate that the reduction of the electron effective mass inside the hydrostatically tensile-strained core of GaAs/In_0.37_Al_0.63_As core/shell nanowires causes a significant enhancement of mobility. The relative increase of mobility with respect to unstrained nanowires and bulk GaAs was measured by OPTPS equal to 30%. A larger increase of 50% is predicted, if the scattering of electrons by photo-generated holes (which is an inherent effect in OPTPS) is excluded, as it would also be the case for high-electron-mobility transistors. It is anticipated that even larger mobility values can be achieved in nanowires with larger lattice mismatch (higher In-content in the shell). The epitaxial growth of In_*x*_Ga_1-*x*_As nanowires with high *x* would be a more direct approach to obtain high electron mobility with III-arsenides on Si, but this is challenging (excluding Au-catalysed methods as incompatible with Si CMOS) because of either the limited incorporation of In into the nanowire crystal in Ga-catalysed growth^57,58^ or the high density of stacking faults in selective area growth^59^. The distinct enhancement of charge carrier mobility shown here is of major importance for the realisation of transistors with high speed and low-power consumption, having the potential to trigger major advancements in high-performance nanowire electronic devices. It is also important that GaAs is anyway among the semiconductors with the highest electron mobilities and that GaAs nanowires can be monolithically integrated in heterogeneous platforms like Si CMOS. Nevertheless, the findings from this work are not only relevant for GaAs, but can also give important guidelines for strained core/shell nanowires made from other semiconductor systems.

## Methods

### Optical-pump THz-probe spectroscopy

The setup is based on a Ti:sapphire femtosecond amplifier (Coherent RegA) operating at a repetition rate of 250 kHz. The pulse duration is ~60 fs and the central wavelength is 800 nm (photon energy of 1.55 eV). The broadband THz-probe pulses are generated using a large-area photoconductive GaAs emitter biased by a modulated voltage and focused onto the sample by an off-axis parabolic mirror with a focal length of 10 cm. The FWHM THz spot size is estimated to be 0.7–0.8 mm. The probe pulses transmitted through the sample are detected via electro-optic sampling in a (110) ZnTe crystal. The optical-pump beam is modulated by an optical chopper and focused by a lens at a point located far behind the sample. The resulting pump spot FWHM size at the sample is 1.7 mm—more than the doubled THz spot size. In this way, we ensure homogeneous photoexcitation conditions. The photo-induced change in the transmitted THz field is measured using a double demodulation technique by two lock-in amplifiers.

### Time-resolved photoluminescence spectroscopy

The excitation source is a Ti:sapphire laser at 1.55 eV photon energy. The fluence is ~8 μJ cm^−2^ with a spot diameter of 20 μm. The pulse length is 2 ps. The photoluminescence is collected with a 100× objective and dispersed in a spectrometer (100 grooves mm^−1^). The photoluminescence is measured with a streak camera with a time resolution of 9 ps.

### Reporting Summary

Further information on research design is available in the Nature Research Reporting Summary linked to this article.

## Supplementary information

Supplementary Information

Lasing Reporting Summary

## Data Availability

The data that support the findings of this study are available from the corresponding author upon reasonable request.
