# Supplementary material for: High electron mobility in strained GaAs nanowires
Source: Nat Commun. 2021 Nov 17;12:6642. doi: 10.1038/s41467-021-27006-z (PMC8599471; doi:10.1038/s41467-021-27006-z)
Supplement: Supplementary file 1 — Supplementary Information [file 41467_2021_27006_MOESM1_ESM.pdf]

SUPPLEMENTARY INFORMATION

High electron mobility in strained GaAs nanowires

Leila Balaghi,[1, 2] Si Shan,[1] Ivan Fotev,[1, 2] Finn Moebus,[1] Rakesh Rana,[1] Tommaso Venanzi,[1, 2] René Hübner,[1] Thomas Mikolajick,[2, 3] Harald Schneider,[1] Manfred Helm,[1, 2] Alexej Pashkin,[1] Emmanouil Dimakis[1, *]

[1] Institute of Ion Beam Physics and Materials Research, Helmholtz-Zentrum Dresden-Rossendorf, 01328 Dresden, Germany

[2] Centre for Advancing Electronics Dresden (cfaed), Technische Universität Dresden, 01062 Dresden, Germany

[3] NaMLab gGmbH, Dresden, Germany

[*] Contact information: e.dimakis@hzdr.de

Supplementary Note 1. Compositional and dimensional analysis of GaAs/In_xAl_{1-x}As core/shell nanowires

The chemical composition and the dimensions of GaAs/In_xAl_{1-x}As core/shell nanowires were measured by scanning transmission electron microscopy (STEM) and energy-dispersive X-ray spectroscopy (EDXS).

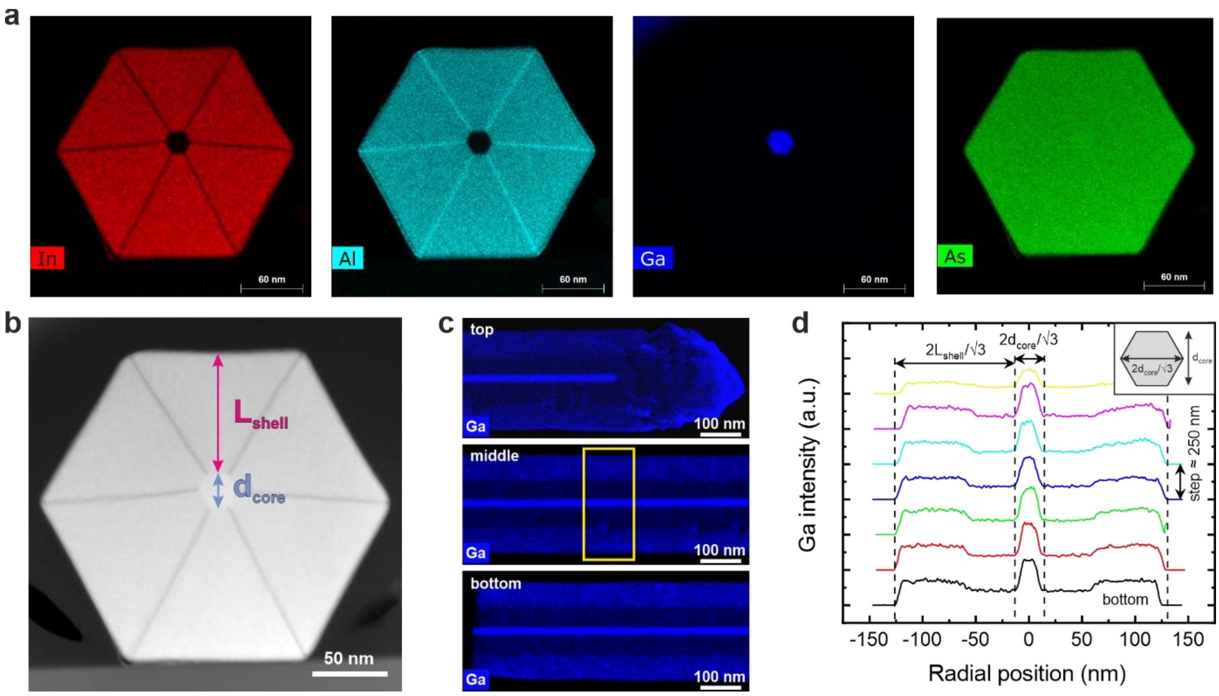

Figure S1. Measurement examples of core and diameter compositions and thicknesses in GaAs/In_xAl_{1-x}As core/shell nanowires using cross-sectional (a, b) or side-view (c) STEM/EDXS analysis. In-, Al-, Ga-, and As- intensities are shown in red, cyan, blue, and green colour, respectively. (d) Radial profiles of Ga-intensity (zero position at the centre of the core) at different positions along the nanowire axis (step distance ≈ 250 nm). The intensity was integrated using rectangular areas (yellow frame in (c)).

Supplementary Note 2. Numerical calculation of stress and strain in GaAs/$In_xAl_{1-x}As$ core/shell nanowires using finite-element continuum elasticity

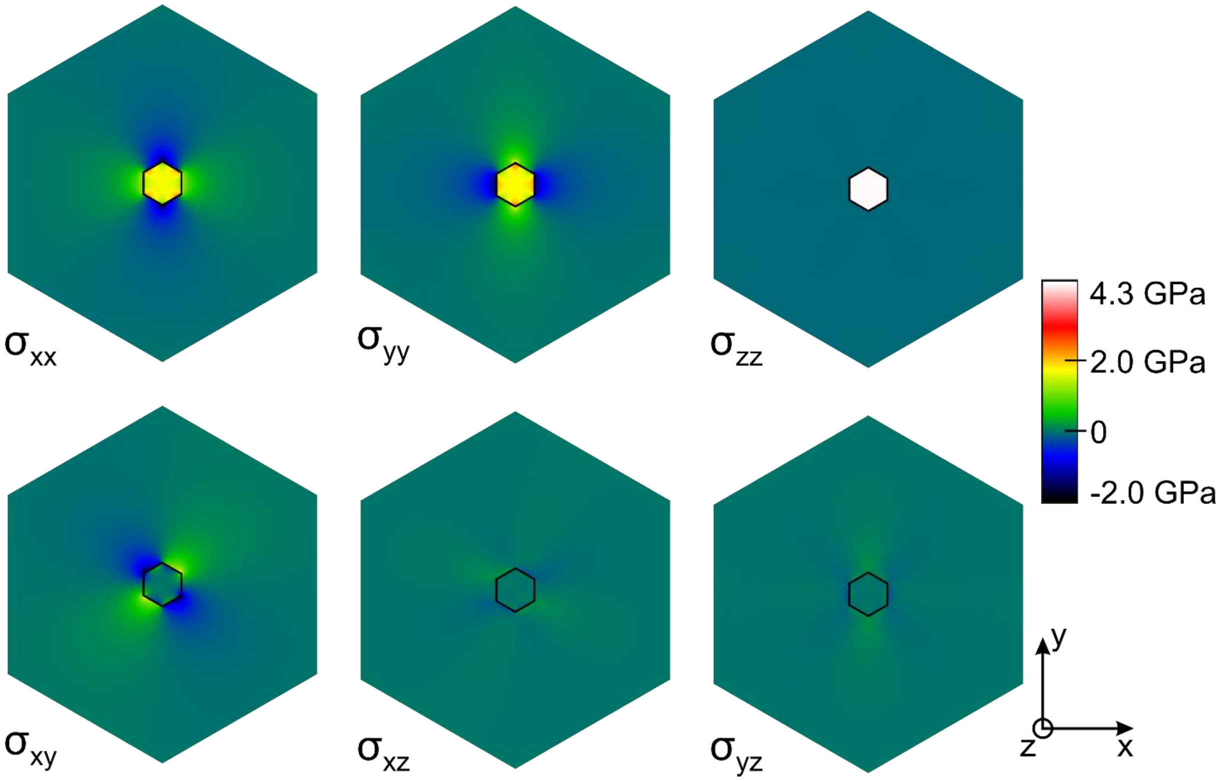

Figure S2-1. Simulated stress components close to the core of a GaAs/$In_{0.37}Al_{0.63}As$ core/shell nanowire using continuum elasticity theory. The nanowire axis is parallel to the z-direction. The core has a diameter of 22 nm and is outlined with a black line. The shell is 80 nm thick.

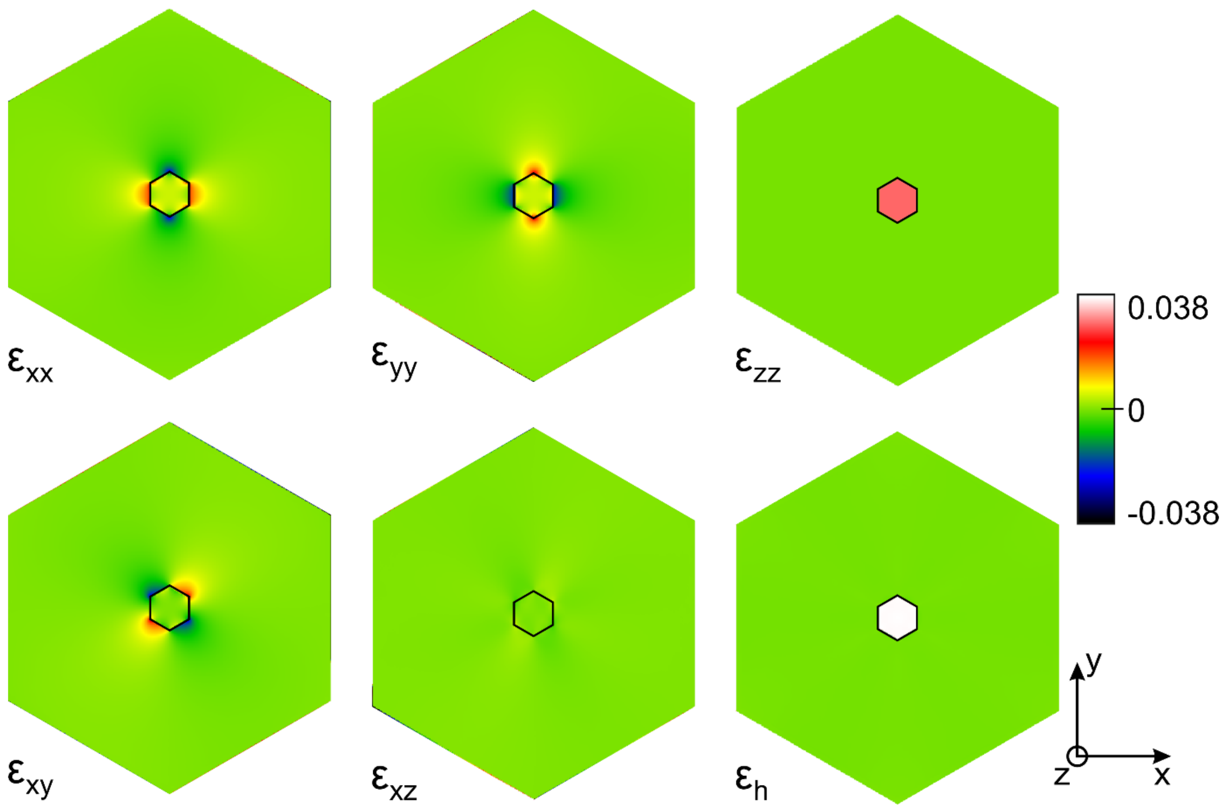

Figure S2-2. Simulated strain components close to the core of a $GaAs/In_{0.43}Al_{0.57}As/In_{0.44}Ga_{0.56}As$ core/shell/capping-shell nanowire using continuum elasticity theory. The nanowire axis is parallel to the z-direction. The core has a diameter of 22 nm and is outlined with a black line. The shell is 80 nm thick and the capping shell is 5 nm thick.

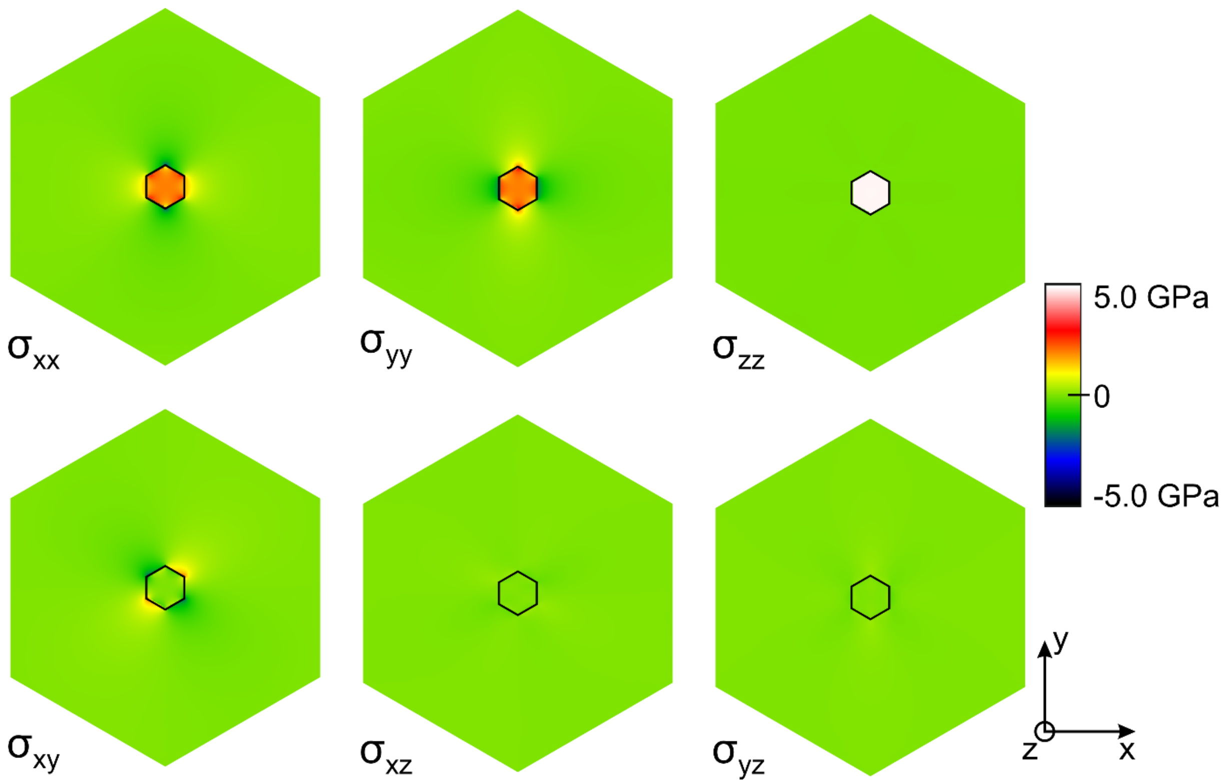

Figure S2-3. Simulated stress components close to the core of a GaAs/$In_{0.43}Al_{0.57}As$/$In_{0.44}Ga_{0.56}As$ core/shell/capping-shell nanowire using continuum elasticity theory. The nanowire axis is parallel to the z-direction. The core has a diameter of 22 nm and is outlined with a black line. The shell is 80 nm thick and the capping shell is 5 nm thick.

Supplementary Note 3. Statistical analysis of the nanowire orientation on OPTPS samples

The transfer method that we used (rubbing of quartz against the as-grown sample) resulted in a common orientation for most of the nanowires. A representative example is shown in Figure S3a. The degree of orientation was evaluated using the “Directionality” plug-in of the Fiji software. The result for Figure S3a is shown in Figure S3b. As the THz field is horizontal (E_{THz} // φ = 0°), a nanowire oriented at an angle φ experiences its projection on the nanowire axis that is proportional to cos φ. We found an angular spread of ±21.5° (using the Gaussian fit as shown by the blue line in Figure S3b), which feels 93% of the incident THz field. The effective contribution of the nanowires in the green shaded region to the plasmon spectral weight is 80% [Rana et al., Nano Lett. 20, 3225–3231, (2020)]. Therefore, the orientation degree of our nanowire samples was sufficient for the observation of the plasmonic response across different set of nanowire samples.

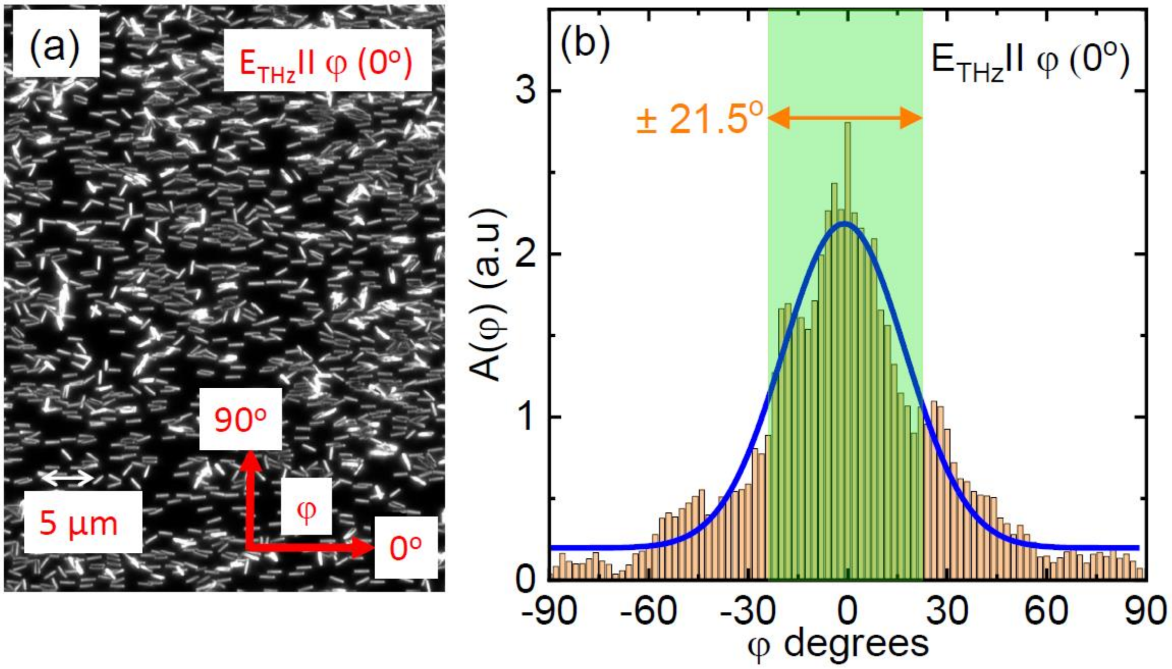

Figure S3. a) Optical microscopy image taken in the dark-field mode of the nanowires on a quartz substrate. The terahertz field ($\mathbf{E_{THz}}$) is applied at an angle φ = 0°; (b) The histogram of the angular nanowire distribution, where the blue line is the Gaussian fit.

Supplementary Note 4. Time resolved photoluminescence measurement on $GaAs/In_{0.43}Al_{0.57}As/In_{0.44}Ga_{0.56}As$ core/shell/capping-shell nanowires.

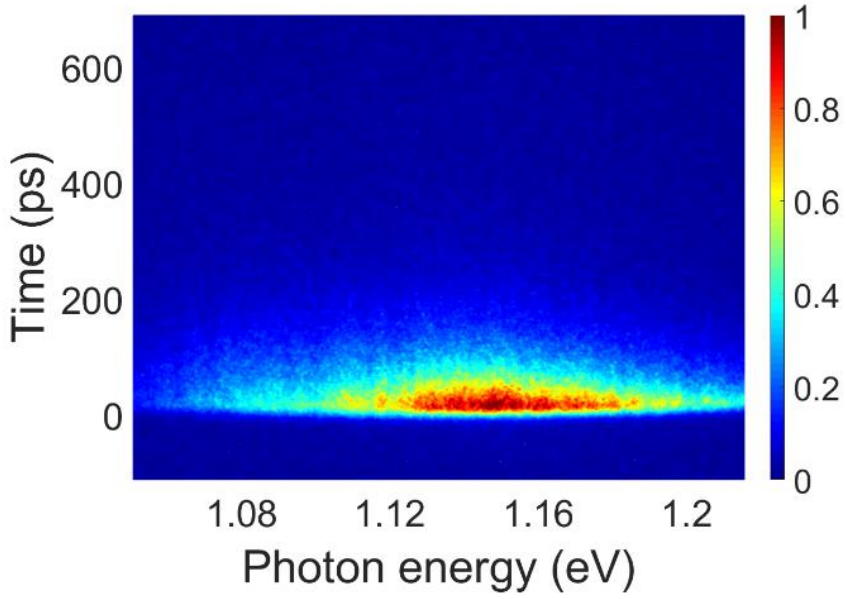

Figure S4. Time-resolved photoluminescence spectroscopy scan from capped $GaAs/In_{0.43}Al_{0.57}As/In_{0.44}Ga_{0.56}As$ nanowires at room temperature.
