# Supplementary material for: High electron mobility in strained GaAs nanowires
Source: Nat Commun. 2021 Nov 17;12:6642. doi: 10.1038/s41467-021-27006-z (PMC8599471; doi:10.1038/s41467-021-27006-z)
Supplement: Supplementary file 2 — Lasing Reporting Summary [file 41467_2021_27006_MOESM2_ESM.pdf]

nature research

Corresponding author(s): Emmanouil Dimakis

Lasing Reporting Summary

Nature Research wishes to improve the reproducibility of the work that we publish. This form is intended for publication with all accepted papers reporting claims of lasing and provides structure for consistency and transparency in reporting. Some list items might not apply to an individual manuscript, but all fields must be completed for clarity.

For further information on Nature Research policies, including our data availability policy, see Authors & Referees.

ü Experimental design

Please check: are the following details reported in the manuscript?

1. Threshold

   Plots of device output power versus pump power over a wide range of values indicating a clear threshold
   ☐ Yes
   ☒ No
   Not relevant

2. Linewidth narrowing

   Plots of spectral power density for the emission at pump powers below, around, and above the lasing threshold, indicating a clear linewidth narrowing at threshold
   ☐ Yes
   ☒ No
   Not relevant

   Resolution of the spectrometer used to make spectral measurements
   ☐ Yes
   ☒ No
   Not relevant

3. Coherent emission

   Measurements of the coherence and/or polarization of the emission
   ☐ Yes
   ☒ No
   Not relevant

4. Beam spatial profile

   Image and/or measurement of the spatial shape and profile of the emission, showing a well-defined beam above threshold
   ☐ Yes
   ☒ No
   Not relevant

5. Operating conditions

   Description of the laser and pumping conditions
   Continuous-wave, pulsed, temperature of operation
   ☐ Yes
   ☒ No
   Not relevant

   Threshold values provided as density values (e.g. W cm^{-2} or J cm^{-2}) taking into account the area of the device
   ☐ Yes
   ☒ No
   Not relevant

6. Alternative explanations

   Reasoning as to why alternative explanations have been ruled out as responsible for the emission characteristics
   e.g. amplified spontaneous, directional scattering; modification of fluorescence spectrum by the cavity
   ☐ Yes
   ☒ No
   Not relevant

7. Theoretical analysis

   Theoretical analysis that ensures that the experimental values measured are realistic and reasonable
   e.g. laser threshold, linewidth, cavity gain-loss, efficiency
   ☐ Yes
   ☒ No
   Not relevant

8. Statistics

   Number of devices fabricated and tested
   ☐ Yes
   ☒ No
   Not relevant

Statistical analysis of the device performance and lifetime (time to failure)

☐ Yes
☒ No

Not relevant
